# The Effect of Anti-Inflammatory and Antimicrobial Herbal Remedy PADMA 28 on Immunological Angiogenesis and Granulocytes Activity in Mice

**DOI:** 10.1155/2013/853475

**Published:** 2013-06-24

**Authors:** Dorota M. Radomska-Leśniewska, Piotr Skopiński, Marcin Niemcewicz, Robert Zdanowski, Sławomir Lewicki, Janusz Kocik, Ewa Skopińska-Różewska, Wanda Stankiewicz

**Affiliations:** ^1^Department of Histology and Embryology, Center for Biostructure Research, Warsaw Medical University, Chałubinskiego 5, 02-004 Warsaw, Poland; ^2^Military Institute of Hygiene and Epidemiology Pulawy, Lubelska 2 Str., 24-100 Pulawy, Poland; ^3^Department of Regenerative Medicine, Military Institute of Hygiene and Epidemiology, Kozielska 4, 01-163 Warsaw, Poland; ^4^Department of Pathology, Center for Biostructure Research, Chałubinskiego 5, 02-004 Warsaw, Poland; ^5^Department of Microwave Safety, Military Institute of Hygiene and Epidemiology, Kozielska 4, 01-163 Warsaw, Poland

## Abstract

PADMA 28 is a herbal multicompound remedy that originates from traditional Tibetan medicine and possesses anti-inflammatory, antioxidant, antimicrobial, angioprotecting, and wound healing properties. The aim of the present study was to evaluate the influence of this remedy on immunological angiogenesis and granulocytes metabolic activity in Balb/c mice. Mice were fed daily, for seven days, with 5.8 mg of PADMA (calculated from recommended human daily dose) or 0.085 mg (dose in the range of active doses of other herbal extracts studied by us previously). *Results*. Highly significant increase of newly formed blood vessels number in *ex vivo* cutaneous lymphocyte-induced angiogenesis test (LIA) after grafting of Balb/c splenocytes from both dosage groups to F1 hybrids (Balb/c × C3H); increase of blood lymphocytes and granulocytes number only in mice fed with lower dose of remedy; and significant suppression of metabolic activity (chemiluminescence test) of blood granulocytes in mice fed with higher dose of PADMA. *Conclusion*. PADMA 28 behaves as a good stimulator of physiological angiogenesis, but for this purpose it should be used in substantially lower doses than recommended by producers for avoiding the deterioration of granulocyte function.

## 1. Introduction

PADMA 28 is a multicomponent, traditional Tibetan herbal plant remedy comprised of 20 specific herbs and 2 nonherbal ingredients. The main PADMA's active substances are bioflavonoids, tannins, phenolic acids, phenolic alcohols, and terpenoids [1, 2]. Weseler et al. presented evidence of antimicrobial activity of this remedy. Both aqueous and alcohol-based PADMA 28 preparations exhibited evident antibacterial effects against Gram-positive bacteria and *Klebsiella pneumonia in vitro *[3]. From 20 herbs present in PADMA, 13 have well-documented antimicrobial activity. *Azadirachta indica* exhibited activity against Gram-positive bacteria. *Aegle marmelos* fruit expresses antifungal activity. *Elettaria cardamomum* (cardamom) essential oil has activity against *Bacillus subtilis* spores. This herb also presents anti-inflammatory and immunotropic activity, enhancing Th2 and suppressing Th1 cytokine release by lymphocytes. Recently, chemopreventive effects of cardamom on chemically induced skin carcinogenesis in mice were described. *Sida cordifolia*, known for its regenerating properties, is active against *Corynebacterium diphtheria* and in combination with nystatin and clotrimazole exhibited antimicrobial effects against five *Candida *strains. Terpenoid eugenol present in *Syzygium aromaticum *expresses general antimicrobial effect; phenolic alcohol from *Glycyrrhiza glabra* was active against *Mycobacterium tuberculosis*, *Staphylococcus aureus,* and *Plasmodium *[2, 4–9]. Recently, anti-influenza viral effects of nuclear export inhibitors from *Valerianae radix* were described [10].

Another PADMA 28 component, *Saussurea lappa* root, and its active principle dehydrocostus lactone inhibit prostate cancer cell migration *in vitro* and have been shown to have anticancer activity. Santamarin, a sesquiterpene lactone isolated from this herb, represses LPS-induced inflammatory response in murine macrophages and potently inhibits the growth of *Trypanosoma brucei rhodesiense* [11–15]. Another PADMA 28 component, *Terminalia chebula* Retz., is called the “King of Medicine” in Tibet. The plant possesses multiple activities, among them, antioxidant, antimicrobial, anti-inflammatory, and wound healing activities [16].

Some immunotropic activities of PADMA 28 and beneficial effect of this remedy in experimental models of inflammation and wound healing were reported [17–24].

In humans, PADMA 28 has been used as a beneficial tonic for heart and blood vessels and as an antioxidant. PADMA 28 has been registered in Switzerland since 1977 by Intercantonal Office for the Control of Medicines as a remedy to alleviate symptoms of claudication, impaired peripheral circulation, pain on walking, leg cramps, and paresthesia. A profitable influence of PADMA 28 was also observed in patients with atherosclerosis and in patients with multiple sclerosis [25, 26]. In 1992, PADMA 28 was registered in Poland. Its efficacy was further proved in prophylactics and treatment of some disorders with inflammatory, sclerotic, and degenerative origins. Treatment of chronic infective pulmonary diseases studied in Poland in a big group of children with PADMA has brought positive results [27, 28]. However, as PADMA is being used for a variety of diseases and usually for a long time (e.g., couple of weeks) and because it possesses strong antioxidative properties, it would affect various parameters of immune system, among them, oxidative burst of granulocytes. In fact, some authors reported that this remedy inhibited the respiratory burst of human neutrophils *in vitro* [29, 30]. That is why we decided to evaluate in the present study, on the experimental model in mice, the *in vivo* effect of PADMA 28 (in high dose, comparable to that recommended for humans, and in low dose, comparable to these which we used previously in studies of various other herbal extracts) on immunological angiogenesis and, simultaneously, on granulocytes metabolic activity evaluated by chemiluminescence. This test is widely accepted as a method of measuring granulocytes oxygen-dependent killing potential. It is important to know if selected doses of this remedy do not disturb granulocytes activity and stimulate immunological angiogenesis and, accordingly, could be used as a safe drug for therapeutic angiogenesis in vascular and immune system disturbances.

## 2. Material and Methods

*PADMA 28 tablets* (batch 28/6311, PADMA AG, Suisse), herbal mixture consisting of 22 ingredients: *Aegle marmelos* fruit (20 mg), *Pimenta dioica* fruit (25 mg), *Aquilegia vulgaris* aerial part (15 mg), *Calendula officinalis* flower (5 mg), *Elettaria cardamomum* fruit (30 mg), *Syzygium aromaticum* flower bud (12 mg), *Saussurea lappa* root (40 mg), *Hedychium spicatum* rhizome (10 mg), *Lactuca sativa* leaf (6 mg), *Cetraria islandica* thallus (40 mg), *Glycyrrhiza glabra* root (15 mg), *Azadirachta indica* fruit (35 mg), *Terminalia chebula* fruit (30 mg), *Plantago lanceolata* aerial part (15 mg), *Polygonum aviculare* aerial part (15 mg), *Potentilla aurea* aerial part (15 mg), *Pterocarpus santalinus* wood (30 mg), *Sida cordifolia* aerial part (10 mg), *Aconitum napellus* tuber (1 mg), *Valeriana officinalis* root (10 mg), camphor (4 mg), and calcium sulfate (20 mg).

### 2.1. Animals

The study was performed on 48 female inbred Balb/c mice 6–8 weeks old, weighing about 20 g, and on 24 female F1 hybrids (Balb/c × C3H), 6 weeks old, delivered from the Polish Academy of Sciences breeding colony. PADMA 28 was administered to mice *per os* in daily doses 5.8 mg or 0,085 mg. Higher dose was calculated according to the highest daily dose (6 tablets), recommended for humans (applying the factor 7 for differences between mouse and human in relation to the surface to body mass). Lower dose conforms to the range of active doses of other herbal extracts and their polyphenolic compounds used in our previous experiments [31–35].

### 2.2. Lymphocyte-Induced Angiogenesis Test (LIA)

Balb/c mice were fed with PADMA (5.8 mg or 0.085 mg) by Eppendorf pipette, in 40 *μ*L of water or 40 *μ*L of water (controls), for 7 days, then bled in anaesthesia (ketamine 100 mg/kg and xylazine 10 mg/kg, BIOWET, Pulawy, Poland) and sacrificed by cervical dislocation. Splenocytes were isolated from spleens of Balb/c donors under sterile conditions by straining through stainless sieve and cotton gauze and centrifugation on Histopaque 1077 (Sigma-Aldrich, USA) for 8 min at 400 g in order to remove erythrocytes. Isolated splenocytes were resuspended in Parker culture medium (TC199, BIOMED, Lublin) and pooled within the groups. A local GVH reaction (lymphocyte-induced angiogenesis, LIA test) was performed according to [36] with some modifications [33]. Shortly, spleen cells suspensions were grafted intradermally (1 million cells in 0.05 mL of Parker medium per graft) into F1 (Balb/c × C3H) recipients. Before performing injections, mice were anaesthetized intraperitoneally with 3.6% chloral hydrate (Sigma-Aldrich, USA; 0.1 mL per 10 g of body mass). Both flanks of each mouse were finely shaved with a razor blade; each flank was injected with cells 2-3 times. Cell suspensions were supplemented with 0.05 mL/mL of 0.01% trypan blue in order to facilitate recognition of injection sites later on. Grafted Balb/c splenic lymphocytes recognized C3H antigens and produced many immunological mediators including proangiogenic factors (immunological angiogenesis). In this test, the number of newly formed blood vessels was the measure of  T-cell reactivity. After 72 hours the mice were treated with a lethal dose of Morbital (Biowet, Puławy, Poland). All newly formed blood vessels were identified and counted in dissection microscope on the inner skin surface, using criteria suggested by the authors of the method, at magnification of 6x, in 1/3 central area of microscopic field. Identification was based on the fact that new blood vessels, directed to the point of cells injection, are thin and differ from the background vasculature in their tortuosity and divarications.

Experiment was performed twice (24 Balb/c mice and 24 F1 hybrids as a total).

### 2.3. Estimation of Leukocytes Number and Their Metabolic Activity (Luminol-Dependent Chemiluminescence Test, CL)

Balb/c mice were fed with PADMA (5.8 mg or 0.085 mg) by Eppendorf pipette, in 40 *μ*L of water or 40 *μ*L of water (controls), for 7 days, then bled in anaesthesia (ketamine 100 mg/kg and xylazine 10 mg/kg, BIOWET Pulawy, Poland) from retroorbital plexus and sacrificed by cervical dislocation. CL was measured using the method of Easmon et al. [37] with some modifications [38–40] at room temperature, in scintillation counter (RackBeta 1218, LKB, Sweden). Briefly, samples of 0.05 mL of heparinised blood were diluted 1 : 4 with phosphate buffered saline (PBS, Biomed Lublin, Poland) and supplemented with 0.1% bovine serum albumin (BSA, Sigma-Aldrich, USA) and 0.1% glucose (Polfa, Poland). Next, 0.05 mL of this diluted blood was mixed with 0.2 mL of luminol (Sigma-Aldrich, USA) solution (10^−5 ^M) in PBS and placed in a scintillation counter in the “out of coincidence” mode for background chemiluminescence measurement. Then, the cells were activated by the addition of 0.02 mL solution of opsonized zymosan (10 mg/mL, Serva, USA), and chemiluminescence activity was measured for the next 15 min. Counting of leukocytes and blood smears examination were performed by routine methods, and the results were shown as the maximum value of chemiluminescence (cpm) obtained for 10^3^ granulocytes. Experiment was performed twice (24 mice as a total).

For all experiments, animals were handled according to the Polish law on the protection of animals and NIH standards. All experiments were accepted by the Local Ethical Committee.

### 2.4. Statistical Analysis

 Statistical evaluation of the results was performed by one-way ANOVA, and the significance of differences between the groups was verified with a Bonferroni multiple comparison post test (Graph Pad Prism software package).

## 3. Results

**Figure 1:**
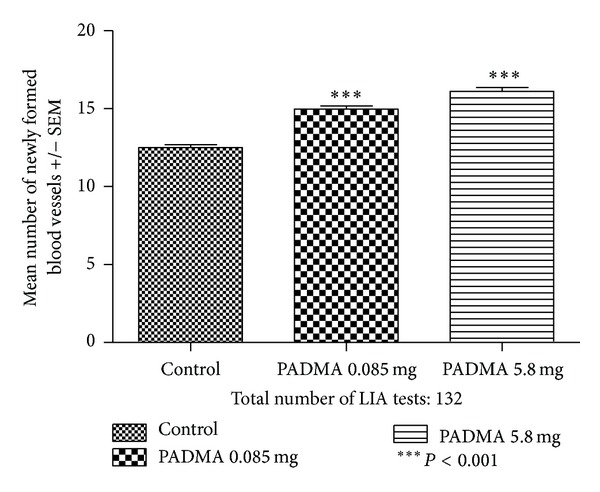
Effect of PADMA 28 (0.085 or 5.8 mg) supplementation of Balb/c donors on the number of newly formed blood vessels measured by lymphocyte-induced angiogenesis test (LIA) in F1 (Balb/c × C3H) recipient mice. Balb/c mice were fed daily *per os* with 0.085 mg or 5.8 mg of PADMA 28. After 7 days, mice were bled and sacrificed, and splenocytes were isolated. Splenocytes were pooled within the groups, resuspended in Parker culture medium (1 million of cells in 0.05 mL), and injected intradermally (5-6 injections per mouse) to 24 F1 (Balb/c × C3H) hybrids. After 72 hours the mice were euthanized, and newly formed blood vessels were counted in dissection microscope (6x magnification). Results are shown as mean ± SEM. ***P < 0.001.

The effect of PADMA 28 (0.085 or 5.8 mg) supplementation of Balb/c donors on the angiogenic ability of their splenic lymphocytes to induce newly formed blood vessels in the skin of F1 (Balb/c × C3H) recipient mice is presented in Figure 1. According to one-way analysis of variance the *P* value <0.0001 is considered extremely significant. Variation among column means is significantly greater than expected by chance. Bonferroni multiple comparison test revealed that PADMA 28 in both doses highly significantly stimulated lymphocytes angiogenic activity as compared to the control and that this stimulation was better after higher dose of remedy (for comparison of lower and higher doses, *P* < 0.01). 

**Figure 2:**
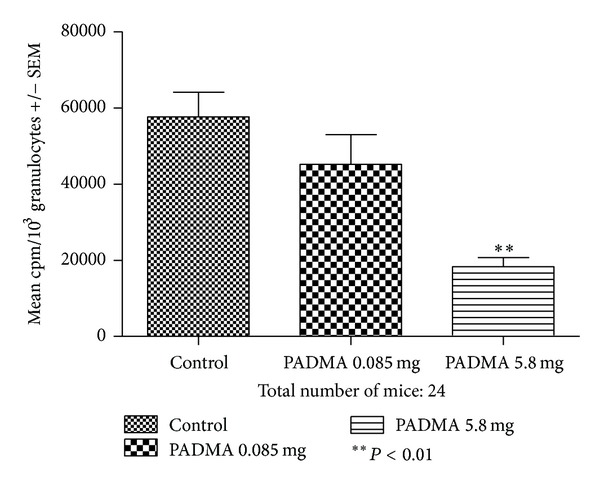
Effect of PADMA 28 (0.085 or 5.8 mg) supplementation on chemiluminescent activity of mouse granulocytes. Balb/c mice were fed daily *per os* with 0.085 mg or 5.8 mg of PADMA 28. After 7 days mice were bled and sacrificed. The chemiluminescence was measured with luminol after activating with blood with zymosan, in scintillation counter (RackBeta 1218, LKB, Sweden). Results are presented as the mean cpm +/− standard error (SEM) per 1000 granulocytes. **P < 0.01.

The results of granulocytes chemiluminescence are presented in Figure 2. According to one-way analysis of variance, the *P* value 0.0019 is considered as significant. Bonferroni multiple comparison test revealed significantly lower chemiluminescence of blood leukocytes collected from mice fed with higher PADMA 28 dose, as compared to the control. 

**Figure 3:**
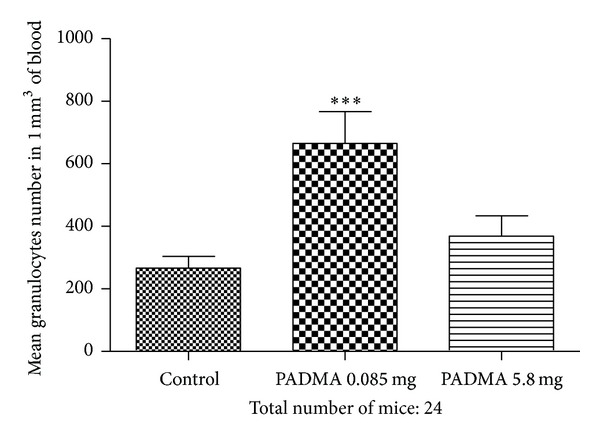
Effect of PADMA 28 (0.085 or 5.8 mg) supplementation on the number of blood granulocytes in mice. Balb/c mice were fed daily with 0.085 mg or 5.8 mg PADMA 28. After 7 days mice were bled and sacrificed. Counting of leukocytes and blood smears examination were performed by routine methods. ***P < 0.001.

The results of PADMA 28 feeding on the blood granulocytes number are presented in Figure 3. According to one-way analysis of variance the *P* value 0.0006 is considered as highly significant. Bonferroni test revealed highly significantly statistical increase of blood granulocytes number in the blood collected from mice fed with lower PADMA 28 dose in comparison to the control (*P* < 0.001) and significant increase (*P* < 0.05) in comparison to the group fed with high dose of remedy. No difference was observed between this last group and control mice.

**Figure 4:**
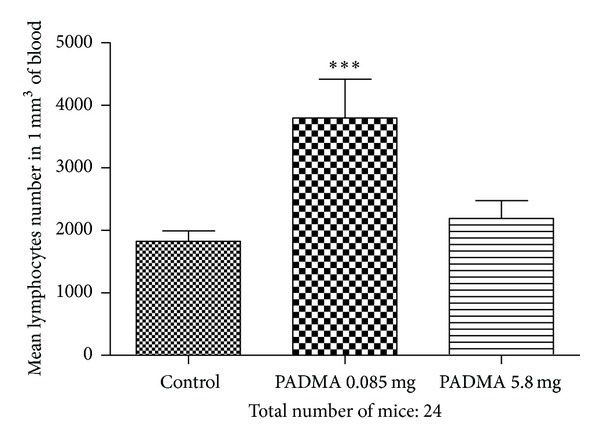
Effect of PADMA 28 (0.085 or 5.8 mg) supplementation on the number of blood lymphocytes in mice. Balb/c mice were fed daily with 0.085 mg or 5.8 mg of PADMA 28. After 7 days mice were bled and sacrificed. Counting of leukocytes and blood smears examination were performed by routine methods. ***P < 0.001.

Number of blood lymphocytes is presented in Figure 4. According to ANOVA, the *P* value 0.0011 is considered as significant. Bonferroni test revealed highly significant increase of blood lymphocytes number in the blood collected from mice fed with lower PADMA 28 dose (*P* < 0.001) in comparison to the control and significant increase (*P* < 0.05) in comparison to the group fed with high dose of remedy. No difference was observed between this last group and control mice.

## 4. Discussion

In this paper, we report for the first time stimulatory effect of multiherbal remedy PADMA 28 on immunological angiogenesis observed in the skin of recipient mice during the local cutaneous graft-versus-host reaction. In our previous studies we obtained similar effects, when we administered to donor mice some other herbal extracts (from plants *Echinacea purpurea*, *pallida*, and *angustifolia* and *Rhodiola rosea*, *quadrifida*, and *kirilowii*) and remedies Echinasal and Bioaron C. [31–35]. However, no stimulatory effect was obtained after feeding donor mice with extract from *Centella asiatica* or multicomponent herbal remedy PERVIVO [31, 41]. On the other hand, inhibitory effect was observed when donor mice were fed with FIBS—an aqueous solution of biogenic stimulators (coastal salt lake mud distillate mixed with cinnamic acid and coumarin) elaborated in 1948 year by Professor V. P. Filatov team [42].

In our present experiments PADMA 28 has behaved as a strong stimulator of proangiogenic factors production by splenic lymphocytes in mice. It remains to elucidate which factors are involved, and this will be the matter of our further studies. It is important that PADMA exerted this angiostimulatory effect also in a substantially lower dose than that recommended by producer, because higher recommended dose significantly inhibited granulocyte respiratory burst measured by chemiluminescence. It should be expected as PADMA 28 contains many compounds demonstrating antioxidant effects—gallic acid, eugenol, ellagitannins, bioflavonoids: quercetin, luteolin, and apigenin, and others [29].

## 5. Conclusion

The ability of PADMA 28 to increase angiogenic activity of lymphocytes may partly explain its beneficial effect on regenerative/repair processes, but for this purpose this remedy should be used in evidently lower doses than these recommended by producers, for avoiding deterioration of granulocyte function.

## References

[1] Gieldanowski J, Dutkiewicz T, Samochowiec L, Wójcicki J (1992). PADMA 28 modifies immunological functions in experimental atherosclerosis in rabbits. *Archivum Immunologiae Et Therapiae Experimentalis*.

[2] Cowan MM (1999). Plant products as antimicrobial agents. *Clinical Microbiology Reviews*.

[3] Weseler A, Saller R, Reichling J (2002). Comparative investigation of the antimicrobial activity of PADMA 28 and selected European herbal drugs. *Forschende Komplementarmedizin und Klassische Naturheilkunde*.

[4] Thangavel M, Raveendran M, Kathirvel M (2006). A comparative study on the effect of plant extracts with the antibiotics on organisms of hospital origin. *Ancient Science of Life*.

[5] Lawrence HA, Palombo EA (2009). Activity of essential oils against *Bacillus subtilis* spores. *Journal of Microbiology and Biotechnology*.

[6] Majdalawieh AF, Carr RI (2010). In vitro investigation of the potential immunomodulatory and anti-cancer activities of black pepper (*Piper nigrum*) and cardamom (*Elettaria cardamomum*). *Journal of Medicinal Food*.

[7] Quiblawi S, Al-Hazimi A, Al-Mogbel M, Hossain A, Bagchi D (2012). Chemopreventive effects of cardamom (*Elettaria cardamomum*L.) on chemically induced skin carcinogenesis in Swiss albino mice. *Journal of Medicinal Food*.

[8] Arif T, Bhosale JD, Kumar N (2009). Natural products: antifungal agents derived from plants. *Journal of Asian Natural Products Research*.

[9] Quedraogo M, Konate K, Lepenque AN, Souza A, ’Batchi B M, Sawadogo LL (2012). Free radical scavenging capacity, anticandicidal effect of bioactive compounds from *Sida cordifolia* L, in combination with nystatin and clotrimazole and their effect on specific immune response in rats. *Annals of Clinical Microbiology and Antimicrobials*.

[10] Watanabe K, Takatsuki H, Sonoda M, Tamura S, Murakami N, Kobayashi N (2011). Anti-influenza Vidal effects of novel nuclear export inhibitors from *Valerianae Radix* and *Alpinia galanga*. *Drug Discoveries and Therapeutics*.

[11] Kim EJ, Hong JE, Lim SS (2012). The hexane extract of *Saussurea lappa* and its active principle, dehydrocostus lactone, inhibit prostate cancer cell migration. *Journal of Medicinal Food*.

[12] Wang C-Y, Tsai A-C, Peng C-Y (2012). Dehydrocostuslactone suppresses angiogenesis *in vitro* and *in vivo* through inhibition of AKT/GSK-3*β* and mtor signaling pathways. *PLoS ONE*.

[13] Choi HG, Lee DS, Li B, Choi YH, Lee SH, Kim YC (2012). Santamarin, a sesquiterpene lactone isolated from *Saussurea lappa* represses LPS-induced inflammatory responses via expression of heme oxygenase-1 in murine macrophage cells. *International Immunopharmacology*.

[14] Pandey MM, Rastogi S, Rawat AKS (2007). *Saussurea costus*: botanical, chemical and pharmacological review of an ayurvedic medicinal plant. *Journal of Ethnopharmacology*.

[15] Julianti T, Hata Y, Zimmermann S, Kaiser M, Hamburger M, Adams M (2011). Antitrypanosomal sesquiterpene lactones from *Saussurea costus*. *Fitoterapia*.

[16] Bag A, Bhattacharyya SK, Chattopadhyay RR, Rashid RA (2013). The development of *Terminalia chebula* Retz. (*Combretaceae*) in clinical research. *Asian Pacific Journal of Tropical Biomedicine*.

[17] Badmaev V, Kozlowski PB, Schuller-Levis GB, Wisniewski HM (1999). The therapeutic effect of an herbal formula Badmaev 28 (padma 28) on experimental allergic encephalomyelitis (EAE) in SJL/J mice. *Phytotherapy Research*.

[18] Barak V, Kalickman I, Halperin T, Birkenfeld S, Ginsburg I (2004). PADMA-28, a Tibetan herbal preparation is an inhibitor of inflammatory cytokine production. *European Cytokine Network*.

[19] Aslam MN, Warner RL, Bhagavathula N, Ginsburg I, Varani J (2010). A multi-component herbal preparation (PADMA 28) improves structure/function of corticosteroid-treated skin, leading to improved wound healing of subsequently induced abrasion wounds in rats. *Archives of Dermatological Research*.

[20] Exner M, Raith M, Holzer G, Gmeiner B, Wagner O, Kapiotis S (2006). Anti-inflammatory mechanisms of the Tibetan herbal preparation Padma 28 in the vessel wall. *Forschende Komplementarmedizin*.

[21] Ginsburg I, Rozenstein-Tsalkovich L, Koren E, Rosenmann H (2011). The herbal preparation padma 28 protects against neurotoxicity in PC12 cells. *Phytotherapy Research*.

[22] Moeslinger T, Friedl R, Volf I, Brunner M, Koller E, Spieckermann PG (2000). Inhibition of inducible nitric oxide synthesis by the herbal preparation Padma 28 in macrophage cell line. *Canadian Journal of Physiology and Pharmacology*.

[23] Wójcicki J, Samochowiec L, Kadlubowska D (1989). Inhibition of ethanol-induced changes in rats by PADMA 28. *Acta Physiologica Polonica*.

[24] Skopiński P, Radomska-Leśniewska DM, Sokolnicka I, Bałan BJ, Siwicki AK, Skopińska-Różewska E *In vivo* stimulatory effect of Multi-component herbal remedy PADMA 28 on mitogen-induced proliferation of mice splenic lymphocytes and their chemokinetic activity.

[25] Korwin-Piotrowska T, Nocon D, Stankowska-Chomicz A, Starkiewicz A, Wojcicki J, Samochowiec L (1992). Experience of Padma 28 in multiple sclerosis. *Phytotherapy Research*.

[26] Melzer J, Brignoli R, Saller R (2006). Efficacy and safety of padma 28 in peripheral arterial occlusive disease. *Forschende Komplementarmedizin*.

[27] Jankowski A, Jankowska R, Brzosko WJ (1992). Treatment of children prone to infection with PADMA 28. *Schweizerische Zeitschrift für Ganzheitsmedizin*.

[28] Jankowski A, Lewandowicz-Uszynska A, Leszczyk-Kapusta I, Borowiec A (1999). The use of immunostimulation in children with recurrent respiratory tract infections. *Nowiny Lekarskie*.

[29] Ginsburg I, Sadovnik M, Sallon S (1999). PADMA-28, a traditional Tibetan herbal preparation inhibits the respiratory burst in human neutrophils, the killing of epithelial cells by mixtures of oxidants and pro-inflammatory agonists and peroxidation of lipids. *Inflammopharmacology*.

[30] Matzner Y, Sallon S (1995). The effect of Padma-28, a traditional Tibetan herbal preparation, on human neutrophil function. *Journal of clinical & laboratory immunology*.

[31] Skopińska-Rözewska E, Furmanowa M, Guzewska J, Sokolnicka I, Sommer E, Bany J (2002). The effect of *Centella asiatica*, *Echinacea purpurea* and *Melaleuca alternifolia* on cellular immunity in mice. *Central-European Journal of Immunology*.

[32] Siwicki AK, Skopińska-Rózewska E, Hartwich M (2007). The influence of *Rhodiola rosea* extracts on non-specific and specific cellular immunity in pigs, rats and mice. *Central-European Journal of Immunology*.

[33] Skopińska-Rózewska E, Wójcik R, Siwicki AK (2008). The effect of *Rhodiola quadrifida* extracts on cellular immunity in mice and rats. *Polish Journal of Veterinary Sciences*.

[34] Skopińska-Rózewska R, Wasiutynski A, Sommer E (2011). Modulatory effect of *Echinacea pallida* on cellular immunity and angiogenesis in mice. *Central European Journal of Immunology*.

[35] Skopińska-Rózewska E, Wasiutyński A, Skopiński P, Siwicka D, Zdanowski R, Bodera P (2011). *In vivo* effect of two complex herbal remedies Echinasal and Bioaron C on antibody production and immunological angiogenesis in mice. *Central-European Journal of Immunology*.

[36] Auerbach R, Kubai L, Sidky Y (1976). Angiogenesis induction by tumors, embryonic tissues, and lymphocytes. *Cancer Research*.

[37] Easmon CSF, Cole PJ, Williams AJ, Hastings M (1980). The measurement of opsonic and phagocytic function by luminol-dependent chemiluminescence. *Immunology*.

[38] Cohen HJ, Newburger PE, Chovaniec ME, Whitin JC, Simons ER (1981). Opsonized zymosan-stimulated granulocytes: activation and activity of the superoxide-generating system and membrane potential changes. *Blood*.

[39] Tono Oka T, Ueno N, Matsumoto T (1983). Chemiluminescence of whole blood. 1. A simple and rapid method for the estimation of phagocytic function of granulocytes and opsonic activity in whole blood. *Clinical Immunology and Immunopathology*.

[40] Skopińska-Rózewska E, Bychawska M, Sommer E, Siwicki AK (2008). The *in vivo* effect of *Rhodiola quadrifida* extracts on the metabolic activity of blood granulocytes in mice. *Central-European Journal of Immunology*.

[41] Balan BJ, Stankiewicz W, Skopinska-Różewska E (2013). The effect of multi-component herbal remedy PERVIVO on cellular immunity and tumor angiogenesis in mice. *Central European Journal of Immunology*.

[42] Skopiński P, Sommer E, Skopińska-Różewska E (2001). The effect of coastal salt lake mud distillate mixed with cinnamic acid and coumarin (FIBS) on immunological and inflammatory angiogenesis. *Terapia*.

